# Light/Clock Influences Membrane Potential Dynamics to Regulate Sleep States

**DOI:** 10.3389/fneur.2021.625369

**Published:** 2021-03-29

**Authors:** Masashi Tabuchi, Kaylynn E. Coates, Oscar B. Bautista, Lauren H. Zukowski

**Affiliations:** Department of Neurosciences, Case Western Reserve University School of Medicine, Cleveland, OH, United States

**Keywords:** circadian clock, sleep, synaptic plasticity, membrane potential, neural coding

## Abstract

The circadian rhythm is a fundamental process that regulates the sleep–wake cycle. This rhythm is regulated by core clock genes that oscillate to create a physiological rhythm of circadian neuronal activity. However, we do not know much about the mechanism by which circadian inputs influence neurons involved in sleep–wake architecture. One possible mechanism involves the photoreceptor cryptochrome (CRY). In *Drosophila*, CRY is receptive to blue light and resets the circadian rhythm. CRY also influences membrane potential dynamics that regulate neural activity of circadian clock neurons in *Drosophila*, including the temporal structure in sequences of spikes, by interacting with subunits of the voltage-dependent potassium channel. Moreover, several core clock molecules interact with voltage-dependent/independent channels, channel-binding protein, and subunits of the electrogenic ion pump. These components cooperatively regulate mechanisms that translate circadian photoreception and the timing of clock genes into changes in membrane excitability, such as neural firing activity and polarization sensitivity. In clock neurons expressing CRY, these mechanisms also influence synaptic plasticity. In this review, we propose that membrane potential dynamics created by circadian photoreception and core clock molecules are critical for generating the set point of synaptic plasticity that depend on neural coding. In this way, membrane potential dynamics drive formation of baseline sleep architecture, light-driven arousal, and memory processing. We also discuss the machinery that coordinates membrane excitability in circadian networks found in *Drosophila*, and we compare this machinery to that found in mammalian systems. Based on this body of work, we propose future studies that can better delineate how neural codes impact molecular/cellular signaling and contribute to sleep, memory processing, and neurological disorders.

## Introduction

Circadian rhythms regulate an endogenous biological clock that dictates a sleep–wake cycle running in ~24-h intervals. These rhythms occur even in complete darkness, but they are reset by light. Over the past 50 years, researchers have elucidated many molecular genetic components that regulate the Light/Clock interactions. Many of these core clock genes were identified with forward mutagenesis screens in *Drosophila*. For example, period (PER) and timeless (TIM) oscillate to create transcription–translation feedback loops (1–3). In addition, cryptochrome (CRY) was identified as a clock-related gene that is sensitive to light and modulates the circadian rhythm in *Drosophila* (4). However, CRY is a central part of the molecular clock in mammals but lacks light sensitivity (5). In *Drosophila*, CRY is an important element that communicates with the light/clock integrator (6), and it influences the neural activity of circadian clock neurons (7) by interacting with potassium ion channel β-subunit redox sensor (8).

In addition to CRY signaling, other core clock output molecules modulate the neural activity of circadian clock neurons by interacting with a number of proteins, such as ligand-gated channels (9, 10), voltage-dependent/independent channels (11), channel-binding protein (12), and subunits of the electrogenic ion pump (12). In *Drosophila*, synaptic plasticity that regulates sleep was induced by specific sequences of spikes that occur during spontaneous activity in clock neurons (12). Moreover, based on recent studies, the interaction of light and clock information influences memory learning, possibly mediated by sleep (13, 14). These studies made an interesting link between light and clock information in forming neural coding, which is based on “neural activity” of circadian clock networks.

When neurophysiological researchers wish to quantify “neural activity,” they often assess action potential firing (or spiking), which is typically shown as mean firing rate (15, 16), first spike latency (17), relative spike timing (18), or regularity of interspike intervals (19–23). This definition is based on the concept, in which neurons represent information with sequences of spikes (24, 25). However, a number of “non-spiking neurons” do not generate action potentials (26–29). Instead, they can transmit information with a temporal structure of subthreshold membrane potential (30, 31). Researchers question whether neurons and spiking activity are the basic units of brain function. This model is based on the prediction that neural network signals flow along well-behaved axonal rails and pass the activity baton at synapses.

**Figure 1:**
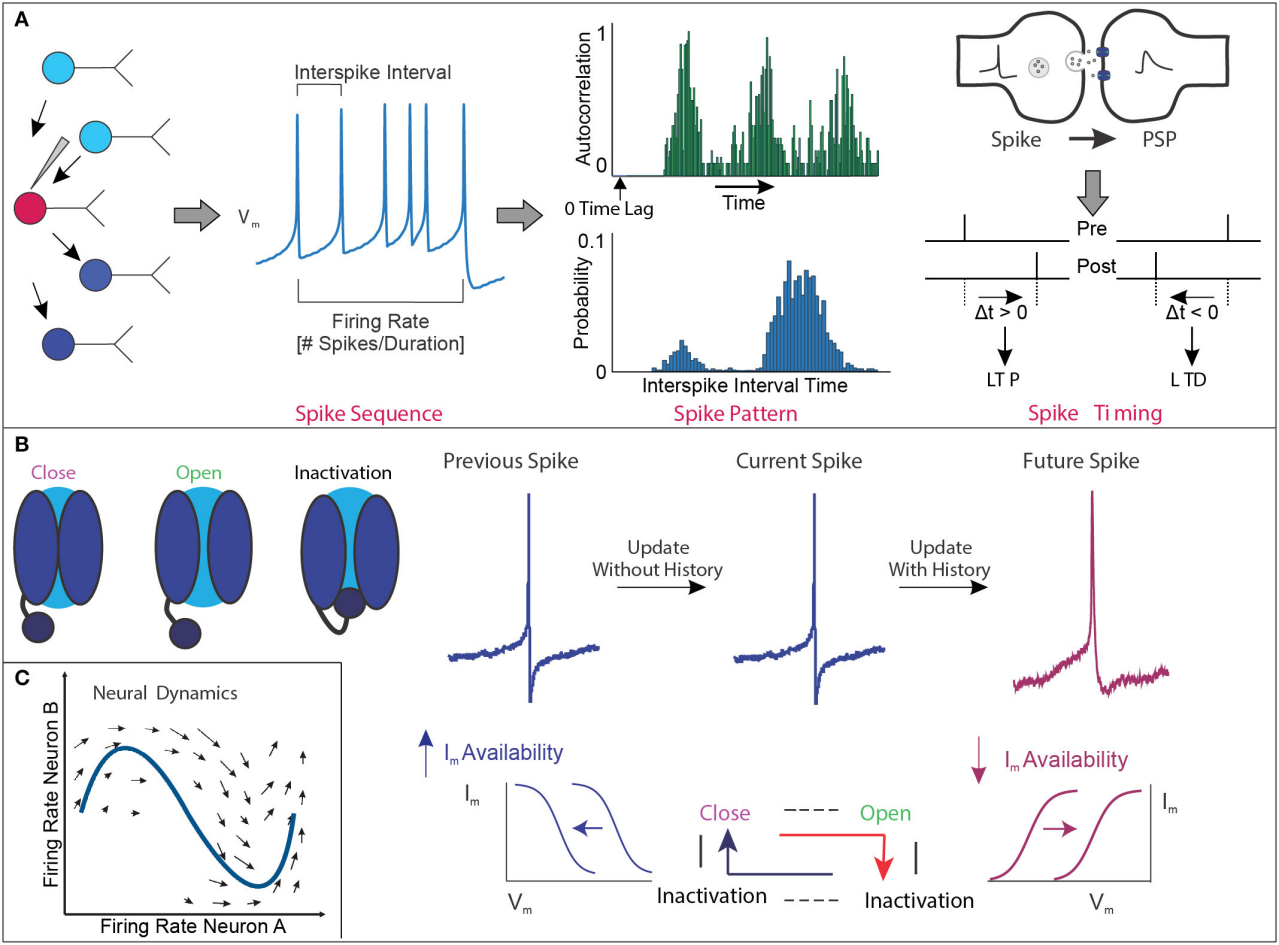
Membrane potential dynamics. **(A)** Conceptual example of the quantification of membrane potential (Vm) dynamics representing the widely accepted temporal structure of the action potential spike sequence obtained during electrophysiology recordings. These analyses include the mean frequency of spikes within a given amount of time, regularity of spiking based on the probability distribution of the interspike interval, and autocorrelation function of spike trains over time to assess patterned neural timescales, as well as relative timing between presynaptic spike and postsynaptic spike to generate LTP/LTD. Note that the conceptual example focuses on the Vm of single cells and not a population of cells. **(B)** Conceptual example of hierarchical biophysical interactions between single channels and Vm state change to generate the hysteresis effect in time. In the presence of “perfect deactivation of inactivation” of voltage-gated ion channels following spike generation, availability of net ionic currents (Im) is increased, making set point shifting of Im–Vm relation (shown in blue). In this situation, there is no update of history in the next spike generation event, and thus each spike generation event is independent. In the presence of “imperfect deactivation of inactivation,” availability of Im is decreased, making set point shifting of Im–Vm relation (shown in magenta). Decreased availability of Im makes “updated history” in the next spike generation event, and thus, the next spike generation event is significantly influenced by the previous event. These sequential chains shape hysteresis effect to generate the higher-order temporal structure of spike trains. **(C)** Conceptual example of neural activity in the activity space. As the firing frequency of the two neurons changes, the neural activity moves in this two-dimensional space. If we observe the activity of *n* neurons at the same time, the activity pattern can be described in an *n*-dimensional activity space.

Regardless of whether spiking activity is used for information representation, the “membrane potential dynamics” of neurons is the essence of neuronal information representation (32, 33). In this review, we define membrane potential dynamics as the stochastically/deterministically shaped temporal structure of membrane potential (34, 35) (Figure 1). The significance of membrane potential dynamics can be shaped by changes in hierarchical biophysical interactions (33), from microscopic [e.g., thermal noise (36), single-channel dwell time variability (37–39)] to mesoscopic [e.g., synaptic conductance variability (40–45)] to macroscopic [e.g., the hierarchical interplay of multiple neurons (46–49)] changes.

In this review, we propose that membrane potential dynamics produced by circadian photoreception and core clock molecules are critical for generating synaptic plasticity based on the fixed point of neural coding. In this way, these dynamics drive the formation of architecture that support baseline sleep, light-driven arousal, and processing of memory. We will also discuss the machinery that regulates the circadian rhythm and organizes internal (e.g., intrinsic channel conductance) and external (e.g., synaptic conductance) membrane excitability in *Drosophila*, and we will compare this machinery to that found in mammalian systems.

## The Neural Network that Regulates the Circadian Clock

**Figure 2:**
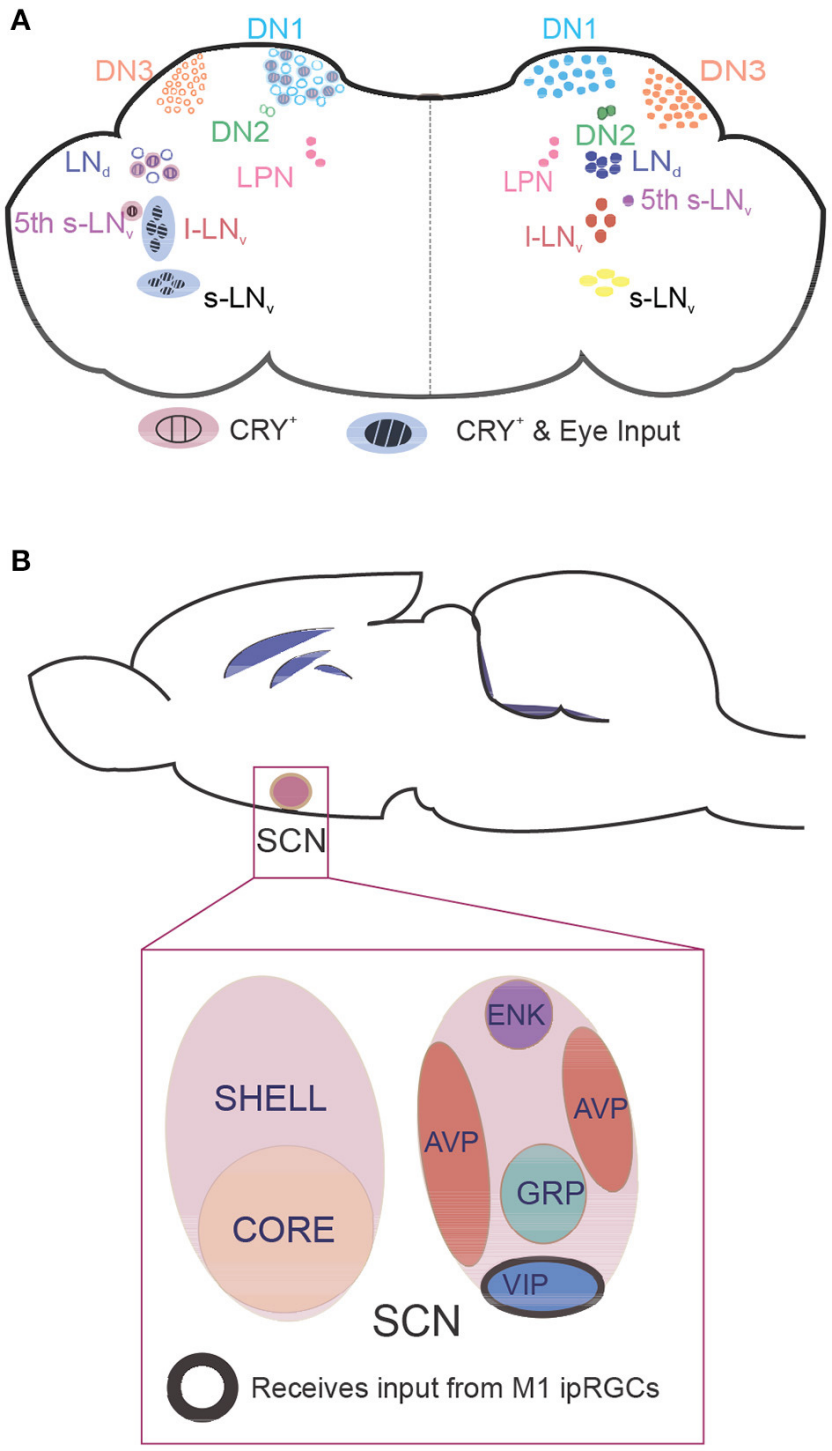
Location of clock neurons in fly and mouse brains. Neurons that express core clock proteins localize in specific regions of the cortex. **(A)** In flies, ~150 clock neurons can be generally categorized as dorsal neurons (DNs) or lateral neurons (LNs). The right hemisphere contains clock neuron populations, and the left hemisphere shows whether these neurons receive retinal input and/or are intrinsically photosensitive due to CRY expression. **(B)** In mammals, core clock neurons are found in the suprachiasmatic nucleus (SCN). Neurons in the photosensitive SCN core primarily express the neuropeptides vasoactive intestinal polypeptide (VIP) and gastrin-releasing peptide (GRP), whereas neurons in the SCN shell express arginine vasopressin (AVP) and enkephalin (ENK). M1 intrinsically photosensitive retinal ganglion cells innervate VIP neurons to pass photic information that synchronizes the clock. CRY, cryptochrome; l-LNv, large ventrolateral neuron; LNd, dorsal lateral neuron; LPN, lateral posterior neuron; s-LNv, small ventrolateral neuron.

In mammals, the master of circadian clock networks is the suprachiasmatic nucleus (SCN) (50). The SCN resides in the hypothalamus and is composed of the ventrolateral (VL) core and VL shell (51). In the VL core, SCN neurons are photosensitive (52, 53) and produce arginine vasopressin (AVP) (54) and enkephalin (ENK) (55) (Figure 2B). Conversely, in the VL shell, SCN neurons are not photosensitive (53), and they express vasoactive intestinal peptide (VIP), gastrin-releasing peptide (GRP), and calretinin (50, 56). Functionally, SCN neurons in the VL core attain visual information relayed from the retina through the retinohypothalamic tract. This relay occurs while SCN neurons in the VL shell gather information from many nuclei, including the hypothalamus and brainstem (56). Interestingly, Pennartz et al. suggest that SCN neurons belong to two classes, class I and class II, that each display spontaneous firing rates (57). In this way, these neurons, which are regulated by calcium-dependent potassium currents, are likely the pacemaker cells of the SCN (58–61). Thus, the varying firing frequencies between the two classes of SCN neurons may influence neuropeptide release to entrain the circadian rhythm and regulate oscillations in circadian-relevant genes.

Neurons within the SCN have expansive heterogeneity, making it challenging for researchers to decipher how SCN neurons structure the circadian rhythm in mammals. To study the circadian rhythm and the neurons that control it, many researchers have turned to flies. In *Drosophila*, specialized clock neurons are enriched with core clock genes that cooperatively drive circadian-dependent activity (62). Approximately 150 clock neurons (63) have been found in the central brain of these flies (Figure 2A). These neurons are categorized based on their location: small VL neurons (s-LNvs) and large VL neurons (l-LNvs), dorsal neurons (DN1, DN2, DN3), lateral posterior neurons, and dorsal lateral neurons (LNds) (64). Although *Drosophila* have fewer neurons than mammals, their circadian clock neurons similarly express many different neuropeptides. Importantly, only half of clock neurons express CRY, yet all clock neurons are synchronized with the light–dark cycle.

To induce daily behavioral rhythms, each subgroup of clock neurons has a specific role in the circadian clock network: the neuronal activity of each subgroup fluctuates in a daily rhythm, peaking at specific times of day and, collectively, their synchronized activity shapes the circadian locomotor behavior. In flies, certain subsets of neurons anticipate morning and evening locomotor activity (65). The s-LNvs, in particular, are the “master clock cells” that entrain the entire circadian rhythm and are responsible for driving morning anticipation in which flies increase their locomotor behavior activity before sunrise. Similarly, the LN_ds_ and l-LNvs, and 5th PDF^−^ s-LNv (with CRY and not PDF) anticipate evening activity (66). Interestingly, DN1s expressing CRY could also contribute to this process (67). DN1s are an upstream target for s-LNvs and LNds, and they modulate sleep–wake patterns (68) by signaling through wake-promoting calcitonin gene-related peptide (a homolog of diuretic hormone 31 in flies) (69) and sleep-promoting glutamatergic connections to s-LNvs (70). Posterior DN1s (DN1ps) are temperature-sensitive neurons that integrate light intensity and temperature to drive evening anticipation (71). Inhibition of these neurons does not affect the delay of the siesta offset at warm temperatures or the decrease of night sleep, implying that these neurons do not promote sleep at warm temperatures (72). This suggests that, in addition to the endogenous clock mechanisms, multimodal sensory inputs can integrate and entrain the circadian rhythm [for DN1p review, see (73)].

In flies, sleep has been behaviorally characterized as consolidated immobility (74) [also reviewed in (75)]. After sleep deprivation, flies exhibit homeostatic recovery of lost sleep (76). Sleep homeostasis is facilitated by brain regions including the mushroom body (MB) (77, 78), dorsal fan-shaped body (FB) (79, 80), and ellipsoid body (EB) (81–83). These structures are controlled by clock neurons and by each other through a variety of neurotransmitters (84). The MB comprises neuropil structures with Kenyon cells that promote wakefulness and sleep, depending on which lobes are affected (77). Serotonin (85) and GABA signaling (86) can inhibit wake-promoting MB neurons. On the other hand, MB output neurons can have a wake-promoting effect by using glutamate (87).

The FB comprises sleep-promoting ExFl2 neurons (88, 89). These neurons can be activated by clock neurons through glutamatergic inputs originating from Allatostatin A-expressing lateral posterior neurons (80). They can also be inhibited by clock neurons to promote sleep through dopamine signaling (79, 90) from the posterolateral cluster 1 and protocerebral posteromedial 3 neurons. To promote sleep, neurons in the FB integrate both types of inputs to signal wake or sleep activity (89).

Concerning sleep–wake regulation, the EB has been implicated to use a D1-like dopamine receptor (91). The EB comprises ring neurons that receive visual information from tubercular bulbar neurons in the anterior visual tract (92). One type of ring neuron, ring layer 5 (R5) neurons (92), may act as an integrator circuit for sleep homeostasis because of their high sensitivity to sleep and persistent upregulation of N-methyl-D-aspartate receptors (NMDARs) (81). R5 neurons exhibit state changes in the form of burst firing, which correlates with slow-wave oscillations (93), which synchronize the overall neuronal activity in this brain region. In addition to correlative activity, the R5 neuron network can use slow-wave oscillations (93) to directly increase the internal sleep drive through NMDARs (81).

The R5 neuron network also integrates information that supports sleep homeostasis by indirectly signaling to ExF12 neurons (81). In response to sleep need, ExF12 neurons induce a mechanism that augments calcium levels, upregulates NMDARs, increases the size and amount of active zones, and amplifies burst firing activity (81). These data support the idea that sleep information can be integrated in the sleep centers of the insect brain. Furthermore, these mechanistic networks may be highly conserved in mammalian circuitry (94–97).

**Figure 3:**
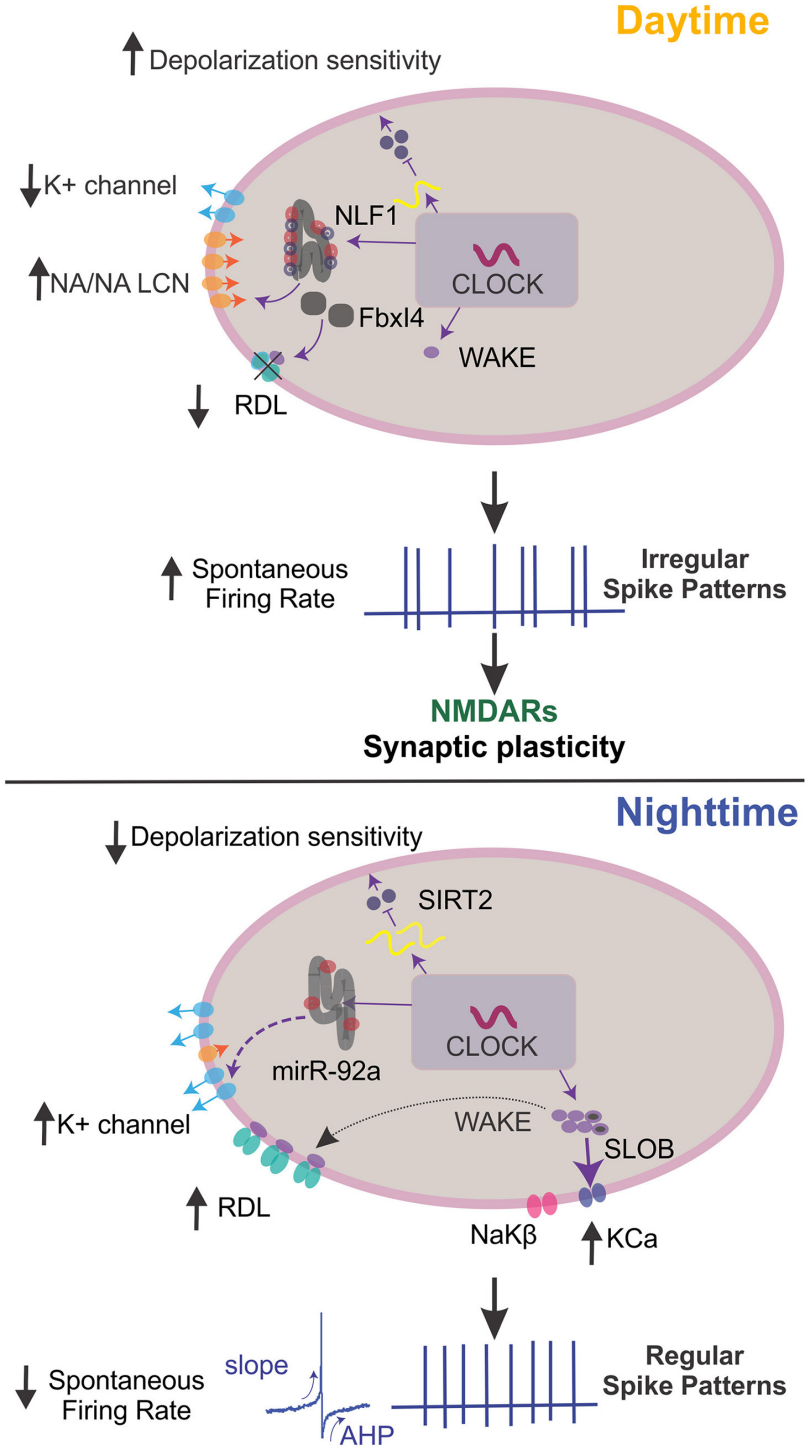
Mechanisms of clock output genes that regulate neuronal excitability. In *Drosophila*, clock output genes modulate membrane potential dynamics to regulate sleep states. During the day, the clock drives high NCA localization factor 1 (NLF-1) activity. This activity, along with narrow abdomen (NA)/sodium leak channel non-selective protein (NALCN) in the lumen of the endoplasmic reticulum, enhances the activity of sodium leak channels and reduces potassium leak conductance to increase action potential (AP) firing rates, vice versa at night. During the night, microRNA-92a (miR-92a) regulates neuronal excitability by inhibiting SIRT2, a NAD-dependent deacetylase of the Sirtuin family and homolog of mammalian *sir2/3*. This inhibition drives a decrease in calcium influx and depolarization sensitivity. At night, wide-awake (WAKE) expression is upregulated and interacts with the GABA_A_ channel Resistance to dieldrin (RDL) to decrease spontaneous AP firing rates and resting membrane potential hyperpolarization. The clock-dependent upregulation of WAKE at night also promotes increased slow-poke binding protein (SLOB) and NaKβ. This process then increases the K_Ca_ current and Na/K ATPase activity, altering spike biophysical dynamics to promote regular firing. When posterior DN1s fire regularly, pars intercerebralis (PI) neurons reduce firing rates, increasing sleep quality. During the day, Fbxl4 is expressed and readily degrades RDL, which increases spontaneous AP rates and burst firing. Also, WAKE is minimally expressed, and clock neurons display irregular firing rates that drive NMDAR-dependent synaptic plasticity in the synapses between DN1p and PI neurons. This change in activity promotes and increases the firing rate in PI neurons, decreasing sleep quality. Note that not all described molecular mechanisms may function in all clock neurons. AHP, afterhyperpolarization.

Clock output molecules have been linked to circadian regulation of sleep through their interaction with neuronal excitability of circadian clock neurons (Figure 3) [reviewed in (98)]. Liu et al. (9) found that the circadian output molecule wide awake (WAKE) responds to CLK oscillations, reducing excitability in wake-promoting l-LN_V_s. This pathway upregulates the GABA_A_ receptor resistant to dieldrin (RDL). They also found that the mammalian homolog of WAKE (9), mWAKE, is expressed in both the SCN and dorsal medial hypothalamus (99). These data support the notion that mWAKE is conserved in driving wakefulness by modulating firing rates.

Conversely, the E3 ubiquitin ligase Fbxl4 downregulates GABA sensitivity in l-LNvs. This downregulation is influenced by RDL ubiquitination, as well as RDL degradation that occurs during the night and peaks at dawn (10). Another study assessed a homolog of the mammalian neuropeptide calcitonin gene-related peptide found in flies (69). This homolog, diuretic hormone 31, was secreted through PDF signaling, increasing neuronal activity to anticipate the morning (68). Additionally, the membrane excitability in DN1p neurons was modulated during the day by the narrow abdomen ion channel, the homolog of the sodium leak channel non-selective protein (NALCN) found in mammals (11). Also, the microRNA mir-92a, which depends on the core clock, was implicated to decrease neuronal excitability in PDF^+^ cells during the night by inhibiting *sirt2* translation (100).

## The Roles of CRY in Flies and Opsins in Mammals

When the clock machinery in *Drosophila* is exposed to light, TIM degradation is the initial response (101), which is mediated by CRY (102). In light, the CRY–TIM complex is bound by JETLAG (JET), which facilitates the ubiquitination and degradation of TIM (103, 104). Once TIM is degraded, CRY binds to JET (105). JET degrades CRY in the presence of light, and the affinity balance between CRY/TIM depends on the two different TIM isoforms, which CRY has stronger affinity to short isoform of TIM (106). Therefore, as light periods progress, decreases in CRY protein reset the molecular clock (107) and augment PER and TIM expression (108, 109). Also, without light, the ubiquitin ligase Cullin-3 regulates circadian control of TIM oscillations (110).

In mammalian systems, light stimulates an electrical response, typically in photoreceptors (111), such as rods and cones. Intrinsically photosensitive retinal ganglion cells (ipRGCs) also participate in photoreception (112, 113). These ipRGCs readily express melanopsin, a blue light-sensitive photopigment encoded by the *Opn4* gene (114, 115). In mammalian models, the cell bodies of ipRGCs reside mostly in the outer nuclear layer (ONL) and 5–14% in the inner nuclear layer (INL) (116). A subset of ipRGCs can also be found in the area of the retinal edge called the ciliary marginal zone, inner plexiform layer (IPL) (116). Six classes of ipRGCs (M1–M6) have been characterized (117, 118), which are mainly dictated by where they stratify in the IPL and the synaptic input from bipolar cells [reviewed in (119)]. For example, M1 ipRGCs stratify in the outer sublamina of the IPL, where they form excitatory synapses with bipolar cells that detect light fluctuations; M2, M4, and M5 cells stratify solely in the inner sublamina; and M3 and M6 cells are bistratified in both layers (118, 120). Additionally, ipRGCs have distinct intrinsic membrane properties (118, 121), light responses (120, 122), and areas in the brain to which they send projections [reviewed in (123, 124)].

All classes of ipRGCs express melanopsin, to some capacity, and most express the Brn3b transcription factor. The only exception is a subclass of M1 ipRGCs (125). These Brn3b-negative neurons send projections to the SCN and the VL geniculate nucleus and intergeniculate leaflet in the thalamus (125, 126). Light processed by Brn3b-negative M1 ipRGCs in the retina project to the SCN via the retinohypothalamic tract, distinct from the canonical visual pathway, and synapse with neurons expressing VIP to mediate light-dependent circadian resetting (127, 128). Brn3b-negative M1 ipRGCs transduce light information to clock cells in the SCN through glutamatergic (129, 130) and GABAergic signaling (131). They also generate receptor potential through hyperpolarization-activated cyclic nucleotide–gated (132) and transient receptor potential cation (133) channels. These melanopsin-expressing ipRGCs are necessary for light processing as mammals cannot respond to external light conditions when melanopsin and rhodopsin in the retina are ablated (134). This effect occurs because circadian activity relies on light input information that the retinal ganglion cells receive and relay through the retinohypothalamic tract to reach the SCN (135).

In *Drosophila*, CRY is expressed throughout the clock network, in photoreceptors in the retina, and peripheral tissues with clock activity (136). However, in mammals, CRY does not have light sensitivity. Instead, it works as a transcriptional regulator (5). In *Drosophila*, the function of CRY has been extensively studied. CRY is expressed in the eyes (137), along with six rhodopsins, and in various populations of clock neurons (138), including s-LNvs, l-LNvs, half of LNds, and some DN1s (Figure 2A). This localization allows CRY to integrate light information by modulating the neuronal firing rate through the redox sensor of the voltage-gated potassium-channel β-subunit hyperkinetic, at least in l-LNvs in *Drosophila* (7, 8). In addition, the light-induced electrical activity of certain clusters of clock neurons is regulated by visual structures (139), suggesting that both intrinsic and extrinsic light signals are processed in clock neurons. These data also suggest an interplay between CRY and rhodopsins, which could be observable in the specific light spectrum of wavelengths (140). However, only when CRY and photoreceptor cells (i.e., all six rhodopsins) are removed do clock neurons exhibit circadian blindness (141) in which light entrainment cannot be achieved. Flies express CRY in the rhabdomeres of the photoreceptor cells, which enhances light responses of the circadian clock (142, 143). Moreover, the role of the seventh rhodopsin (Rh7) in sensitizing the CRY-dependent circadian photoresponse has been recently suggested (144). These findings indicate multiple degrees by which photopigments and eyes cooperatively interpret light information.

Interestingly, CRY is expressed in the EB, in what seems to be R5 neurons (137, 145). Because the activity of R5 neurons cycles, the light/clock-generated coding in these cells may have a multilayered structure that influences the circadian clock network and sleep-drive circuits. This theory is supported by a recent paper showing how light-input controls night sleep at the circuit level, independent of the clock (146). Thus, light information could be an important state variable in the relationship between circadian regulation of sleep and sleep homeostasis.

## Spike Coding in the *Drosophila* Circadian Clock

The rate of action potential firing and changes in neuronal excitability have been observed in clock neurons in both mammals and flies (147). In mammals, SCN neurons exhibit spontaneous and signature firing patterns throughout the day, remain silent at night, increase action potential production at dawn, and maintain a steady firing pattern for the rest of the light period (148, 149). Many changes in SCN neuronal activity can be attributed to the intrinsic membrane currents of sodium and potassium (147). Sodium currents lead to increased excitability in the daytime, and potassium currents create a hyperpolarized membrane potential at night (150–152).

In mammals, voltage-gated potassium channels mediate changes in neuronal membrane dynamics in circadian clock neurons (151, 152). Recently, the *Drosophila* model showed that the interaction of CRY with voltage-gated potassium channels mediated changes in neuronal membrane dynamics (8). These channels help stabilize the membrane potential by maintaining it closer to the potassium equilibrium potential. This mechanism of stabilization occurs alongside terminating fast-acting action potentials and by controlling the interspike interval timing during recurrent neuronal firing (153). Stabilization is also augmented by lowering the membrane's sensitivity to excitatory inputs. Prompted by excitatory inputs, voltage-gated potassium channels undergo several inactivated conformational states (154, 155).

In addition to the steady-state transitions of potassium-channel kinetics, many researchers have proposed models for the non-steady-state kinetic activation and inactivation (156–158) of these voltage-gated potassium channels. They found that there are varying kinetic substates where there are more specific closed and inactivation states within these three simplified states. A variety of substates of voltage-gated channels differentially regulate membrane potential dynamics (159), and the proven interaction between CRY and potassium channels is related to beta-subunit (accessory) but not alpha-subunit (core) function (8). Thus, the overarching role of CRY-mediated signaling may be to modulate the timing of these state transitions, which could be a state variable of membrane potential dynamics. However, further studies are needed to support this speculation. In addition, Agrawal et al. showed an interaction between CRY and potassium channels in salivary glands (160). Although the association between CRY and potassium channels was found without the involvement of light, these data also support the idea that the signaling complex between CRY and voltage-gated potassium channels regulate membrane potential dynamics.

In response to upstream clock signaling, *Drosophila* show daily modulations of neuronal electrophysiological properties in clock neurons. Early work used local field potentials to detect changes in electrophysiological activity during dynamic sleep–wake states (161). Specifically, changes in potassium channels (162–164), sodium channels (39), and other modulatory molecules (165) regulate membrane potential-related changes. When exposed to light, flies use CRY signaling to increase action potential firing in l-LN_VS_ (7). In this process, CRY binds to the Shaker voltage-gated potassium-channel β-subunit channel subunit hyperkinetic (8), a protein implicated in sleep-dependent memory. This binding induces flavin redox-mediated regulation of potassium conductance (8). Additionally, Quasimodo (166), a light-responsive factor, modulates the firing rhythm of clock neurons via Shaw, a Kv3.1 potassium voltage-gated channel, and a sodium-potassium-chloride (Na^+^ K^+^ Cl^−^) cotransporter. Because Quasimodo is involved in multiple components, further studies need to elucidate the interactive mechanism that modulates the membrane potential dynamics of l-LNvs. Shal/Kv4, a voltage-gated potassium channel, was sufficient to modulate sleep–wake transitions by suppressing time-specific rates of neuronal firing (167). Fernandez-Chiappe et al. performed a screen that identified hyperpolarization-activated cation current (168). They showed that this current is important for high-frequency bursting of l-LNvs (168). Apart from molecular modulation of electrophysiological activity in neurons, slow-wave oscillations can confer increased sleep need in flies, further implicating the importance of electrical activity with behavioral states (93). These observations suggest that clock neurons readily manipulate ion conductance by modulating potassium activation–inactivation states to mediate changes in neuronal excitability and behavioral states.

Due to the growing body of literature on circadian clock neurons and changes in neuronal excitability, many new models attempt to explain how circadian oscillations are maintained in relation to changing excitability states. For example, output molecules that interact with core clock genes may reveal insights into the translation of clock information to behavior. Early models proposed that circadian-mediated oscillations in resting membrane potentials are driven by circadian cycles (169–171). Flourakis et al. found that membrane potential, regulated by sodium and potassium currents, is mediated by the circadian clock in *Drosophila* (11). *Drosophila* DN1p membrane potentials create rhythms throughout the day, as well as sodium and potassium conductance rhythms, indicating that resting membrane potentials may be mediated by circadian control. NALCN ion channels facilitate these rhythms in membrane conductance through the oscillatory expression of its localization ER protein Nlf-1 (11). Based on these data, Flourakis et al. propose a “bicycle” model in which two cycles of conductances work in opposed temporal phases. In the morning/day, potassium currents decrease, leading to increased sodium leak facilitated by NA/NALCN and more depolarized membrane potential in DN1p neurons that increases “resting” sodium conductance (172) and their firing rates. The opposite occurs in the evening/night period. This study supports a link between the circadian rhythm and membrane excitability in clock neurons, suggesting that the clock drives molecular rhythms and also physiological changes that maintain robust daily cycles.

Much of the literature deciphers the possible mechanisms through which the circadian clock can modulate neuronal membrane potential. However, fly researchers have started to study clock neurons and their changing excitability as they relate to sleep structure. Recently, Tabuchi et al. (12) questioned whether temporal codes (the timing and pattern of neuronal firing) can induce changes in neuronal firing rates or other related physiological behaviors. This study expanded previous work showing that sensory stimuli can induce temporal codes in target neurons (19). Tabuchi et al. found that DN1p neurons exhibited circadian-dependent spiking patterns with distinct characteristics based on daytime and nighttime settings (12). Specifically, in DN1p neurons, the daytime temporal code consisted of an irregular spike train with a second-order temporal structure, whereas the nighttime temporal code had a more regular pattern. The second-order temporal structure was defined by the probabilistic density of adjacent pairs of interspike intervals. They also found that these temporal spike patterns are generated by WAKE.

Oscillations in WAKE expression mediate the sleep quality by interacting with the calcium-dependent potassium channel (KCa) and a novel sodium/potassium ATPase β subunit, which are upregulated at night under clock and WAKE control. Upregulation of KCa activity leads to a deeper after hyperpolarization of DN1p spikes, which slows firing during periods of increased input. Conversely, sodium/potassium ATPase activity accelerates spike onset, which maintains spiking during periods of reduced input. The combination of increased KCa and sodium/potassium ATPase activity promotes spike morphologies with faster onset and deeper afterhyperpolarization, which leads to regular firing and greater sleep quality at night (Figure 3).

Tabuchi et al. also demonstrated a causal role for temporal coding in sleep behavior. They found that circadian-dependent changes in the spiking pattern of DN1p clock neurons encodes arousal and regulates sleep (12). However, we still do not know how DN1p clock neurons transmit the spiking pattern information to downstream neurons. DN1p clock neurons directly project to an arousal circuit in the pars intercerebralis (PI) to regulate sleep/wake behavior (173, 174). Tabuchi et al. examined how a cyclic spiking pattern in DN1p neurons affects downstream signaling. They discovered that only the irregular DN1p firing pattern increased the downstream PI neuron firing rate. This transmission of DN1p temporal codes to PI neuron rate codes was mediated by a novel form of synaptic plasticity driven solely by the temporal pattern of neural spiking. These observations implicate that temporal spike patterns may modulate behaviors, such as sleep, and induce synaptic plasticity in downstream targets (Figure 3).

These studies further our knowledge about how neurons can modulate output behaviors through changes in physiology. The circadian clock may modulate behavior through multiple mechanisms in which clock-dependent molecules influence membrane excitability and temporal spike codes. More circadian-driven behaviors could be modulated through temporal spiking patterns because clock neurons may use multiple distinct codes. However, further studies are needed to closely assess how environmental light can modulate membrane excitability and temporal spike codes.

## CRY Bridges Light Input and Spike Pattern

CRY is located in PDF^+^ l-LNvs and s-LNvs, as well as LN_ds_ and DN1s (6, 138, 175), where the photoreceptor responds to light pulses by restarting the circadian oscillation of PER and TIM levels. This effect occurs through CRY binding to TIM (176, 177), although it has been reported that TIM degradation for phase delays (which CRY is required) within s-LNvs is neither necessary nor sufficient (178), suggesting a multiplex regulatory system. CRY also influences the rate of action potential firing (7). CRY can modulate membrane depolarization and the action potential firing rate by interacting with and using the Kvß redox sensor hyperkinetic (8). Wang et al. found evidence that mammalian systems have a non-transcriptional pathway for redox modulation under circadian control (179). They found that K^+^ conductance oscillates in SCN neurons, suggesting that neuronal activity can be controlled by protein redox states (179).

As described above (see section The Neural Network that Regulates the Circadian Clock), distinct populations of clock neurons drive the circadian rhythm at different times of day. As evening descends, the driving force of activity becomes the responsibility of LNds in the presence of light, illustrating the rearrangement of neuronal circuits according to the photoperiod (180). Thus, CRY-mediated signaling may control spike temporal coding to signal changes in behavior. Moreover, depending on the zeitgeber time, changes occur in global circuit switching (181), structural plasticity (182), and temporal spike coding in DN1p neurons (12). This drives neuronal patterning to arrhythmicity and PI neurons to activate through NMDAR-mediated synaptic plasticity, ultimately reducing sleep quality (12). Understanding the heterogeneous nature of DN1p could reveal the significance of differences between molecular signatures and activity.

## Proper Memory Consolidation Requires A Functional Clock

The circadian clock may have strong implications in proper memory consolidation. In humans, chronic disruption in the circadian clock has been linked to mild cognitive impairment and even dementia and Alzheimer's disease (183, 184). The search for mechanisms that regulate memory consolidation affected by circadian desynchronization has been challenging in rodent models. Whether clock genes (CRY1 and 2) are genetically knocked out or the SCN is surgically ablated, rodent models seem to have no drastic changes in memory (185). This challenge could mean that for adult-onset memory phenotypes to occur in dysrhythmic animals, the SCN neural network must be intact, both genetically and physically. This theory has been supported by a study in Siberian hamsters. Indeed, Fernandez et al. found that the SCN circuitry must be preserved for an arrhythmic SCN to have deleterious effects on memory (186). They hypothesized that in arrhythmic SCN hamsters, daily GABA signaling from the SCN is disrupted and downstream target memory centers are inhibited (187). This mechanism could also mediate a clock-driven suppression of synaptic plasticity to prime learning centers for continued learning.

The hippocampus has long been implicated in long-term memory (LTM) and may modulate LTM through sleep. Recently, researchers proposed that sharp wave-ripples are important for the consolidation process of LTM that contributes to deficits in emotional memory (188), sequential memory (189), spatial memory (190–192), and synaptic plasticity (193, 194). Interestingly, sleep/wake states can further complicate the consolidation process by modulating sharp wave-ripples through hippocampal pathways (188, 194). These observations suggest that sleep may act upstream of important memory circuits and mechanisms in the hippocampus. Other studies used arrhythmic models to decipher how the circadian clock can further facilitate LTM. They found that the circadian clock modulates synaptic plasticity (195) and spatial memory (187). In addition, the role of astrocytes has recently been suggested. Brancaccio et al. demonstrated that NMDAR expressed in the dorsal SCN is responsible for neuron–astrocyte interactions to suppress SCN neurons during nighttime, suggesting astrocytes control extracellular glutamate circadian cycles to regulate the synchronization of the SCN neural network (196). McCauley et al. (197) also found that astrocytes specific to CA1 hippocampal pyramidal neurons oscillate near these neurons. These astrocytes also cycle NMDAR expression. Ultimately, these changes in receptor expression modify synaptic plasticity to mediate oscillations in hippocampal-dependent learning and implicate an astroglial cell type in modulating circadian-driven changes in behavior. A role of functional connections between astrocytes and l-LNv circadian clock neurons in modulating sleep drive EB in *Drosophila* also supports plastic mechanisms shaped by astrocytes and clock neurons (198).

In *Drosophila*, specific regions of the brain regulate learning and memory pathways. For example, MBs are located in the protocerebrum of the brain, where they are organized into five lobes that process olfactory learning and memory. Furthermore, MBs may also participate in the interplay between sleep regulation and memory centers (87). Due to the downscaled circuitry of the fly brain, a single pair of neurons that function as inputs to MBs, the dorsal paired medial neurons (DPMs), regulate the consolidation of odor memories. DPMs have a dual role in memory consolidation and GABAergic sleep promotion. Specifically, DPMs may inhibit mushroom bodies through GABA and 5-HT signaling to promote memory consolidation and increased sleep (86). Dorsal fan-shaped body neurons (dFBs) also exhibit dual roles in sleep and memory consolidation. In LTM formation, this form of memory consolidation would not occur without thermogenically activated dFB neurons and a courtship training paradigm. Thus, increasing sleep through DPMs and dFBs may simultaneously enhance the power of memory consolidation neurons (199).

Additionally, LTM is also controlled by clock genes, most notably PER. In *Drosophila*, the cAMP–MAPK–CREB pathway may be crucial to memory formation (200). CREB regulates PER expression by binding to an upstream domain, and PER null mutants cannot form LTM after courtship conditioning, indicating that in this context, PER is also crucial in LTM formation (201). In mammals, a similar mechanism may occur. Indeed, sleep deprivation decreases cAMP activity in the hippocampus of mammals, reducing memory consolidation (202). Overall, these studies in mammals and flies strongly suggest that there is a relationship between memory consolidation and circadian rhythms, a relationship that should be further explored.

## Light Plays A Critical Role in Maintaining LTM *via* Circadian Clock Signaling

To understand if sleep is necessary for memory consolidation, many studies have relied on methods involving sleep deprivation, as well as changes in proteome levels, stress levels, and neural activities. Alternatively, researchers have disrupted sleep continuity in a stress-free manner by optogenetically activating hypocretin (also known as orexin) neurons in mice (203). This approach revealed that only a minimal amount of continuous sleep is needed to properly consolidate memories.

Light, or the absence of light, could also maintain robust LTM. In human studies, certain light exposure can affect cortical areas that are important for cognition. For example, differing wavelengths of light affected memory and attention (204–206). In rodent models, varying degrees of light exposure modulate tone-cued and contextual fear conditioning (207, 208). Light exposure was also found to modulate long-term potentiation through ipRGC mechanisms, which has been linked to modulating memory consolidation (209). Although these studies link light and cognitive processing, the tasks evaluated in these studies vary according to the type of memory processing. Also, we still do not know the molecular mechanisms that contribute to the connection between light photoreceptors and memory structures.

Recently, environmental light was implicated as necessary for maintaining LTM through PDF^+^ l-LNvs. Inami et al. used a memory paradigm involving courtship conditioning to assess how consolidated memory is maintained and how environmental light participates in this process (13). In this study, they entrained flies to the paradigm and then placed them in either constant darkness or constant light. They found that flies in constant darkness exhibited impaired LTM, whereas flies in constant light had intact LTM (13). They also found that in flies exposed to constant darkness, LTM was rescued by activation of PDF^+^ neurons. Moreover, in a recent paper, Flyer-Adams et al. showed that PDF is important for regulating olfactory associative memory in *Drosophila* (14). This finding suggests that light/clock-generated information in LTM that is mediated by l-LNvs could be associated with cognitive performance.

## Conclusions

The circadian clock controls both molecular and behavioral rhythms that are needed for many organisms to survive. In this review, we propose that the balance of light (mediated by CRY in Drosophila and melanopsin in mammals) and core clock signaling regulate membrane potential dynamics to modulate synaptic plasticity that depends on neural coding, as well as circuitry, memory, and sleep architecture. Moreover, recent work in *Drosophila* and rodent models uncovered molecular pathways that underlie these changes. In addition, the non-circadian roles for light input and possible implications for brief light treatment therapy have been explored. However, we do not yet have a clear understanding of whether and how CRY can interact with these downstream clock effectors. Finding molecular links between these two mechanisms could reveal other pathways that are restricted to clock neurons and can facilitate circadian-dependent behavior. Future studies are needed to understand the mechanism through which CRY integrates light signals throughout the circadian clock. Importantly, mammalian CRY does not have photosensitivity, which is different from CRY found in *Drosophila*. However, in mammals, the light input system shifts the rhythm via melanopsin-positive ganglion cells in the retina, resulting in sleep–wake effects. Further investigation will help elucidate if the principles of the role of Light/Clock-generated neural coding can be said to be fundamentally conserved between *Drosophila* and mammals.

At the molecular level, computation in the brain starts with proteins, such as voltage-gated ion channels and receptors that can change their structural and functional state in response to environmental changes, such as light. These molecular mechanisms have been adapted into increasingly more complex computational frameworks. In synapses and neurons, local membrane potentials shaped by ion channels and receptors are temporally and spatially integrated. Furthermore, individual neurons are interconnected into networks and circuits, and the circuits are assembled into a brain capable of abstract thought. Not surprisingly, electronic computing has mirrored the same pattern in the engineering world, starting from transistors and integrated circuits to microprocessors and computers. When combined with voltage-gated sodium channels, voltage-gated potassium channels have a crucial function in generating action potentials at the molecular level. To generate action potentials with a specific shape and firing pattern, a neuron needs these voltage-gated ionic channels in appropriate subcellular locations.

How light-induced signaling impacts voltage-gated channels to regulate membrane potential dynamics is unclear. In this review, we propose that a decreased cooperativity of the voltage-gated potassium channel state change timing is vital to generating the higher-order temporal structure of membrane potential dynamics. Mathematically, this state change can be defined as an integrator/differentiator that converts the sum of input signals into an output signal over time, while the output decays steadily. This light-induced state change (mediated by CRY in *Drosophila* and melanopsin in mammals) could be a mechanism that functionally makes some voltage-gated channels unable to simultaneously participate in generating membrane potential dynamics. Therefore, such a decreased cooperativity of these channels must enhance the hysteresis effect and, thus, may lead to the higher-order temporal structure of membrane potential dynamics. Light signaling adds a powerful mechanism for sensing light and controlling the history-dependency of membrane potentials, which is critical for generating specific patterns of spike trains.

As we elucidate clearer connections between light, the circadian clock, and clock-driven behaviors, we can make meaningful efforts to use pharmaceuticals or simply light as a form of treatment against circadian pathologies.

## Author Contributions

All authors listed have made a substantial, direct and intellectual contribution to the work, and approved it for publication.

## Conflict of Interest

The authors declare that the research was conducted in the absence of any commercial or financial relationships that could be construed as a potential conflict of interest.
